# Comparison of laparoscopic and open pyloromyotomy: Concerns for omental herniation at port sites after the laparoscopic approach

**DOI:** 10.1038/s41598-019-57031-4

**Published:** 2020-01-15

**Authors:** Fenne A. I. M. van den Bunder, Ernest van Heurn, Joep P. M. Derikx

**Affiliations:** Emma Children’s Hospital, Amsterdam UMC, University of Amsterdam, Department of Pediatric surgery, Amsterdam, 1100 DD The Netherlands

**Keywords:** Stomach diseases, Medical research

## Abstract

Pyloromyotomy is a common surgical procedure in infants with hypertrophic pyloric stenosis and can be performed with a small laparotomy or laparoscopically. No specific complications have been documented about one of the approaches. We aim to study (severity of) complications of pyloromyotomy and to compare complications of both approaches. Children undergoing pyloromyotomy between 2007 and 2017 were analyzed retrospectively. Complication severity was classified using the Clavien-Dindo classification. We included 474 infants (236 open; 238 laparoscopic). 401 were male (85%) and median (IQR) age was 33 (19) days. There were 83 surgical complications in 71 patients (15.0%). In the open group 45 infants (19.1%) experienced a complication vs. 26 infants in the laparoscopic group (10.5%)(p = 0.013). Severity and quantity of postoperative complications were comparable between both groups. Serosal tears of the stomach (N = 19) and fascial dehiscence (N = 8) occurred only after open pyloromyotomy. Herniation of omentum through a port site occurred only after laparoscopy (N = 6) and required re-intervention in all cases. In conclusion, the surgical complication rate of pyloromyotomy was 15.0%. Serosal tear of the stomach and fascial dehiscence are only present after open pyloromyotomy and omental herniation after laparoscopy respectively. The latter complication is underestimated and requires attention.

## Introduction

Pyloromyotomy, the treatment of infantile hypertrophic pyloric stenosis (IHPS), is one of the most common surgical procedures performed in children. During the procedure the hypertrophied pyloric muscle is transected, resulting in resolution of the gastric outlet obstruction. Complication rates vary between 4.6–12%^1,2^. There are no differences in operative and postoperative complication rates between laparoscopic and open pyloromyotomy^3–6^. The most frequently reported complications are incomplete pyloromyotomy, mucosal perforation and wound infection^1,2,7–10^. Other complications are fascial dehiscence and incisional hernia. So far, no complications have been documented which occur only in either one of the approaches. However, herniation of omentum through a port site or ‘Omental herniation’ (OH), which is a rarely described complication, seems to be specifically for the laparoscopic approach.

OH is described in only a few patients after pyloromyotomy and in a couple of children after pediatric abdominal laparoscopic procedures^11–16^. Six cases of OH through a port site are described after laparoscopic pyloromyotomy^11–13^. In a retrospective analyses of pediatric patients who underwent minimal invasive surgery Chen *et al*. and Paya *et al*. describe respectively two trocar-site hernias with omental prolapse in Memphis, Tennessee and three in Vienna (Austria)^15,16^. Omental prolapse following laparoscopic surgery is noted in adults as well^17–20^. Most of the patients required a reoperation to remove the omentum and to close the fascia.

This study set out to examine the complications of pyloromyotomy with specific attention to OH in a large retrospective cohort analysis of patients treated for IHPS in two tertiary pediatric surgical centers. Additionally, this study will compare the complications of the open and laparoscopic approach for treating infantile hypertrophic pyloric stenosis using the Clavien-Dindo classification to provide insight in the severity of complications.

## Methods

Data from all patients who underwent pyloromyotomy at two tertiary pediatric surgical centers in the Netherlands, between January 2007 and December 2017 was analyzed retrospectively. Patients were identified using the National Health Insurance claims, and selected by diagnostic and treatment codes for IHPS and pyloromyotomy. The diagnosis was made by clinical symptoms, metabolic derangements and ultrasound. Patients who were referred with complications of an incomplete pyloromyotomy after initial surgery in other hospitals, patients with anatomic abnormalities and patients with prior surgery of the gastro-intestinal tract were excluded from analysis. The study protocol (2018.210) was approved by the Medical Ethics Committee and the Clinical Research Board. All methods were performed in accordance with the relevant guidelines and regulations.

### Preoperative care

After admission to the hospital infants received a nasogastric tube and intravenous fluid resuscitation. Hypochloremic metabolic alkalosis or other biochemical abnormalities were normalized. All patients were operated under general anesthesia. Prophylactic antibiotics were not given.

### Surgical procedure

The choice for open or laparoscopic approach was up to the surgeons’ preference. In practice all surgeons performed one of the methods exclusively in all patients.

### Open procedure

Open pyloromyotomy was performed by a semi-circular supra umbilical incision, followed by opening of the fascia and mobilization of the stomach. After pyloromyotomy, the stomach was placed back into the abdominal cavity and the fascia was closed. The skin was closed intracuticular or with steristrips.

### Laparoscopic procedure

For laparoscopic pyloromyotomy a 5-mm trocar was introduced in a small sub-umbilical incision. Under direct vision, a 3-mm trocar was inserted or a stab incision was made to introduce a grasper to stabilize the duodenum. A second stab incision was made to introduce a disposable laparoscopic knife in the epigastric region for incision of the serosa, followed by the use of a 3-mm laparoscopic spreader to complete the pyloromyotomy. The myotomy was considered sufficient when both edges could be moved independently. The stomach was filled with air afterwards to exclude a perforation and to confirm that there was passage of air into the duodenum. After removal of the instruments the incisions were closed with either sutures or steristrips. Closure of the fascia was not routinely performed.

### Postoperative care

Full feeding on demand was started three to six hours after surgery. The patient was discharged after tolerating full feeding and monitored at the outpatient clinic two or three weeks after surgery.

### Data collection and analysis

Hospital files and notes were retrospectively reviewed. Listed were sex, gestational age, weight at birth, comorbidities, age at surgery, type of procedure, operating surgeon, overall complications and their consequences and length of hospital stay. The Clavien-Dindo classification was used to evaluate the severity of surgical complications^21^. Mucosal perforations were defined as a disruption of the mucosa which was either identified at the time of the myotomy and repaired immediately, or became apparent in the postoperative period and was re-explored and repaired. Data was extracted by one of the authors (FVDB) and verified by a six percent random sample check.

### Statistics

All analyses were carried out using SPSS, version 24.0. Normality assumption was assessed using Shapiro-Wilk test and (paired) Kolmogorov-Smirnov test. Data were analyzed using Pearson’s Chi-square test or Fisher’s exact test for categorical data and the Mann-Whitney U test for continuous variables, considering p < 0.05 statistically significant.

## Results

### Patient characteristics

During the study period 480 infants underwent pyloromyotomy. Six patients were excluded from analysis because of initial surgery elsewhere (N = 2), an anatomic malformation (N = 2) and previous abdominal surgery (N = 2). Of the 474 included patients 401 were male and 73 female. Median (IQR) patient age was 33 days (19). There were no significant differences in patient characteristics between open pyloromyotomy (N = 236) and the laparoscopic procedure (N = 238). No procedures were converted. Patient characteristics are summarized in Table 1.

**Table 1:** Baseline characteristics open vs lap.

	Total	Open (N = 236)	Laparoscopic (N = 238)	p-value
Sex (M/F)	401/73	195/41	206/32	0.24
Age (days)	33.0^19^	32.5^16^	35.0^23^	0.28
Term/preterm	422/48*	213/23	209/25	0.74
Birthweight (g)	3440.0 [738]*	3425.0 [720]	3457.5 [787]	0.84

Legend Values are patient numbers or median [Interquartile Range].

*Missing values.

### Overall complications

In five patients no obvious hypertrophic pyloric muscle was found during the procedure. These patients nevertheless underwent pyloromyotomy. There were 83 surgical complications in 71 patients (15.0%) of which 26 occurred peroperative and 57 postoperative.

### Overall peroperative complications

Most common peroperative complications were serosal tears of the stomach (4.0%) and accidental full-thickness division of the pylorus with perforation (1.3%), of which four perforations through the mucosa were located at the duodenal side, one at the gastric side and one at the back of the pylorus. The latter one is either caused by traction during mobilization of the stomach or by a technical error of the pyloromyotomy, while other perforations were directly caused by too deep incision of the pylorus. Four were noted during surgery and two after surgery. When diagnosed during the procedure, the perforation was primary closed by stitches. In only one case a new myotomy was conducted on a different position. Two perforations were missed during the procedure and only diagnosed after operation. In the infant who underwent laparoscopic pyloromyotomy an air insufflation check was performed, but nevertheless the perforation was missed. Both infants required re-operation (0.4%). There were no significant differences between open and laparoscopic pyloromyotomy in regard to accidental mucosal perforation noted both during surgery (p = 1.00) and after surgery (p = 1.00).Two infants developed accidental damage of the skin and liver respectively.

### Overall postoperative complications

The incidence of all complications is summarized in Table 2. Postoperative complications were classified using the Clavien-Dindo classification and varied between grades I and IIIb (Table 3). There were no grade IV or V complications in the study population. Frequent postoperative complications were wound infection (4.4%), fascial dehiscence (1.7%) and OH (1.3%). Fascial dehiscence occurred only in patients undergoing laparotomy (N = 8) and was due to stitches tearing through the fascia (N = 4), caused by infection (N = 2) or broken sutures (N = 1). In one patient no cause was recorded. OH exclusively developed after laparoscopic pyloromyotomy, with an incidence of 2.5%. Patient characteristics of infants with OH are presented in Table 4. OH occurred equally on either side of the abdomen (right N = 3, left N = 3) and even after closure of the fascia. All infants required an intervention to reduce the omentum of whom three patients underwent general anesthesia. In total four infants underwent re-pyloromyotomy. Two patients were treated with pneumodilatation after recurrent stenosis after laparoscopic and open pyloromyotomy respectively, of which the latter was complicated by a latent, covered mucosal perforation caused by the initial surgery. Two patients underwent laparoscopic re-pyloromyotomy after an incomplete laparoscopic pyloromyotomy. The re˗interventions were performed 5 to 60 days after the initial procedure. Three children developed sepsis due to Klebsiella pneumoniae (N = 1), Staphylococcus aureus (N = 1), and one patient lacked information about the specific pathogen. Two infants had a hemorrhage; one was coagulated sufficiently during the procedure and one was diagnosed postoperative by a significant decrease in serum hemoglobin levels and required reoperation with coagulation of active bleedings. The bleeding was caused by transient vitamin K deficiency.

**Table 2:** Number of complications of open vs laparoscopic pyloromyotomy.

	Total (incidence %) (N = 474)	Open (N = 236)	Laparoscopic (N = 238)	p-value
**Complications recognized during surgery**				
Intraoperative no signs of IHPS	5 (1.1)	−	−	−
Accidental mucosal perforation (stitched)	4 (0.8)	2	2	1.000
Serosal tears of the stomach	19 (4.0)	19	0	** <0**.**001**
Hemorrhage	1 (0.2)	0	1	1.000
Lesion of the skin	1 (0.2)	1	0	0.498
Lesion of the liver	1 (0.2)	0	1	1.000
**Complications diagnosed after surgery**				
Overlooked mucosal perforation (reoperation)	2 (0.4)	1	1	1.000
Fascial dehiscence	8 (1.7)	8	0	**0**.**004**
Omental herniation	6 (1.3)	0	6	**0**.**030**
Wound infection	21 (4.4)	14	7	0.114
Redo pyloromyotomy (incomplete/recurrent)	4 (0.8)	1	3	0.623
Incisional hernia	10 (2.1)	4	6	0.751
Sepsis	3 (0.6)	2	1	0.623
Hemorrhage (reoperation)	1 (0.2)	0	1	1.000
Peritonitis	1 (0.2)	0	1	1.000
Subcutaneous hematoma	1 (0.2)	1	0	0.498

**Table 3:** Number of postoperative complications classified by the Clavien-Dindo classification.

	*Total*	*I*		*II*		*IIIa*		*IIIb*	
		open	lap	open	lap	open	lap	open	lap
Mucosal perforation	2*	—	—	—	—	—	—	1	1
Fascial dehiscence	8	2	—	—	—	—	—	6	—
Omental herniation	6	—	—	—	—	—	3	—	3
Wound infection	21	9	5	5	2	—	—	—	—
Redo pyloromyotomy	4	—	—	—	—	—	—	2	2
Incisional hernia	10	4	6	—	—	—	—	—	—
Sepsis	3	—	—	2	1	—	—	—	—
Hemorrhage	1*	—	—	—	—	—	—	—	1
Peritonitis	1	—	—	—	1	—	—	—	—
Subcutaneous hematoma	1	1	—	—	—	—	—	—	—

*Complications which were recognized and treated sufficiently peroperative were excluded.

**Table 4:** Patient characteristics of patients with OH.

Sex	Age (days)	Incisions	Closure of the incision after pyloromyotomy	Herniation	Treatment
♂	30	Stab incision in the right lower abdomen and left upper abdomen	All fascia defects are closed with knotted Vicryl sutures and subcutis of the umbilicus was approximated with Vicryl. Skin defects were closed with Steristrips.	Left upper abdomen	Reduction (twice) under local anesthesia and taped with Steristrips.
♀	29	Stab incision in the right lower abdomen and left upper abdomen	All fascia defects are closed with knotted Vicryl sutures and subcutis of the umbilicus was approximated with Vicryl. Skin defects were closed with Steristrips.	Left upper abdomen	Reduction and taped with Steristrips. Unknown whether local anesthesia was used.
♂	40	Insertion of a 3 mm trocar in the right hemi-abdomen and stab incision in the left hemi-abdomen	Infra umbilical fascia is closed with Vicryl sutures followed by intracutaneous closure with Monocryl. Other defects are closed with Steristrips.	Right hemi-abdomen	Resection and reduction of the herniated omentum under general anesthesia. Closure of the fascia with Vicryl sutures.
♂	52	Insertion of a 3 mm trocar in the right hemi-abdomen and stab incision in the left hemi-abdomen	Infra umbilical fascia is closed with Vicryl sutures followed by intracutaneous closure with Monocryl. Other defects are closed with Steristrips.	Right hemi-abdomen	Reduction under general anesthesia.
♂	34	Insertion of a 3 mm trocar in the right side and stab incision in epigastrio	Infra umbilical fascia is closed with Vicryl sutures followed by intracutaneous closure with Monocryl. Other defects are closed with Steristrips.	Right side	Reduction. Unknown whether local anesthesia was used.
♀	29	Stab incision in the right side and in the left upper abdomen.	All fascia defects are closed with Novosyn sutures. Skin defects are closed with Steristrips.	Left upper abdomen	Reduction under general anesthesia. Closure of the fascia with Novosyn sutures.

### Open vs. laparoscopic pyloromyotomy

In the open group 45 patients experienced complications (19.1%) versus 26 patients (10.5%) in the laparoscopic group (p = 0.013). 26 patients experienced a peroperative complication (5.5%), of which 22 occurred in the open group versus four in the laparoscopic group (p < 0.001). This difference was mainly due to serosal tears in the open group. 48 infants developed one or more postoperative complications (10.1%): 26 versus 22 after the open or laparoscopic procedure respectively (p = 0.522). Comparing the complications following the open and laparoscopic procedure there is a difference in fascial dehiscence (p = 0.004) and serosal tears of the stomach (p < 0.001) to the disadvantage of the open approach as well as a significant difference in OH of the laparoscopic approach (Table 2). Differences between both procedures in postoperative complications classified by the Clavien-Dindo classification are shown in Table 3. Severe complications requiring a re-intervention include fascial dehiscence, OH, duodenal perforation and hemorrhage. Postoperative complications led to pharmacological treatment with antibiotics (Clavien-Dindo grade II) in 7 versus 4 patients after the open and laparoscopic approach and to a re-intervention (Clavien-Dindo grade III) in 9 versus 10 patients in the open and laparoscopic approach respectively.

## Discussion

The aim of this study was to assess the surgical complications of open and laparoscopic pyloromyotomy and to compare both procedures. The overall complication rate of pyloromyotomy in our cohort was 15.0%, of which 26 complications occurred during operation and 57 were postoperative. In previous studies, the complication rate ranges from 4.6 to 12%^1,2^. Safford *et al*. described an even lower total complication rate of 2.7% in their large cohort of 11,003 patients^22^. When comparing the rates of the most frequent complications, our results are equivalent to others with rates of 1.3% for mucosal perforation, 4.4% for wound infection and 1.7% fascial dehiscence in our study series^7,8^. Our total complication rate is relatively high compared to others. However most authors did not report operative complications and we searched for complications in all the operative and postoperative notes next to a database searching. We included all possible complications, except for prolonged postoperative vomiting and time to full feed as these are hard to define and affected by several variables^23^. In our series there were differences in type of complications between open and laparoscopic pyloromyotomy in regard to serosal tears of the stomach, fascial dehiscence and OH. Two systematic reviews and meta-analyses noted no differences between peri- and postoperative complications. Both studies did not specifically mention serosal tears of the stomach, fascial dehiscence and OH, but analyzed different combinations of major (perioperative) complications or all complications together^3,4^.

To classify the severity of the complications of pyloromyotomy we used the Clavien-Dindo classification^21^. This is a widely used classification system for grading severity of adverse events by classifying the complication in grades from I to V according to the required therapy. In our study complications of open and laparoscopic pyloromyotomy varied between grade I and III(b). Especially OH, fascial dehiscence and mucosal perforation were ranked high because of the need of reoperation. The distribution between the open and laparoscopic approach was similar. Unfortunately the Clavien-Dindo classification only includes postoperative complications. Although postoperative events may have more impact on the patients’ well-being, it is interesting to score peroperative events as well given their potential impact on operation time and postoperative care.

Serosal tears of the stomach are rarely described with an incidence of 2.5–4.4%^2,24,25^. This is in line with an incidence of 4.0% in our cohort. This is a typical complication for the open approach because of traction of the pylorus to bring the pylorus extraperitoneal. Siddiqui *et al*. mentioned one gastric serosal tear in the open group when comparing open and laparoscopic pyloromyotomy^24^. In our population it also occurred only in children undergoing open pyloromyotomy. The lesions were sutured or treated expectantly and had no consequences for the postoperative management or recovery. This complication is most likely the result of mobilizing the pylorus through a small supra-umbilical incision and can be avoided by extending the incision.

No significant difference in fascial dehiscence between the open and laparoscopic procedure has been reported previously. We report eight cases of fascial dehiscence (1.7%), which all occurred in the open group. This might be a logical consequence of the relatively large supra-umbilical incision used in the open approach versus the small trocar site incisions of the laparoscopic pyloromyotomy. Van Ramshorst *et al*. noted tearing of sutures through the fascia as the most common cause of fascial dehiscence (29%) in a retrospective review of patients with fascial dehiscence after pediatric abdominal surgery. Other causes were infection (13%), broken sutures (10%) and loose knots (5%)^26^. This is in line with our study results.

OH is a rarely described and underestimated complication, particularly related to the laparoscopic approach. It requires reduction of the omentum and closing of the defect, usually performed under general anesthesia. In a review of the literature we only found a few reports describing OH after pyloromyotomy specifically^11,13^. One of the patients presented only after three weeks with incarcerated omentum^12^. This is in contrast to the other patients and our study population who presented within a couple of days after surgery. A retrospective analysis of children who underwent minimal invasive surgery showed that OH is significantly more frequent in young children (<5 years of age) possibly as a result of the thin abdominal wall and weak abdominal muscles^16^. In our study population OH occurred at both sides of the abdomen and occurred independent of closure of the fascia. Although Cost noted no statistically significant relationship between the development of a hernia and port location, port size or fascial closure status in children undergoing urological laparoscopic surgery, others do recommend fascial closure^16,27,28^. It should be taken into account that the proportional size relationship of the trocar versus the intestines is the same when using large (10 mm) trocars in adults and recommends closure as well^29^. The fact we found patients with OH even after closure of the fascia must be interpreted with caution because it is possible that closure of the fascia was not be properly executed due to the small incision site and thin fascia. Possible causes of the occurrence of OH are adhesion of the omentum to the instruments during their removal from the abdomen, poor muscle relaxation before trocar removal, CO2 pushing abdominal contents through the port sites as the abdomen desufflates and weakening of the fascial closure due to infection^11–13,28^. Unfortunately, due to the relatively small amount of patients with OH, we could not perform a risk analysis. To prevent the occurrence of OH, we advise to split the muscles in the direction of the fibers, to pay attention during removal of the equipment and to close the trocar site at the fascial level even at the stab incision sites.

There are some limitations of our study, which are mainly related to the study design. We depended on documentation carried out in the past, which could be partially incomplete. Patients had a short follow-up period; therefore we may have missed some patients with complications without any symptoms. Furthermore it should be appointed that we focused on surgical complications specifically and did not mention pulmonary and cardiovascular complications. Although these types of complications are very rare, they have to be taken into account. Surgeons should be aware of the risk of OH and prevent the occurrence. Further research should be undertaken to investigate the incidence of OH. The relatively high incidence of OH may necessitate mentioning this when counseling the parents.

In conclusion this study has identified an overall complication rate of pyloromyotomy of 15.0%, varying in severity from grade I to IIIb by the Clavien-Dindo classification. When comparing the open versus laparoscopic approach we found a significant difference of peroperative complications and serosal tears of the stomach in disadvantage of the open group. No difference was found in overall postoperative complication rate, but we showed more children with fascial dehiscence in the open group and more patients with OH in the laparoscopic group. OH is an important complication of laparoscopic pyloromyotomy with an incidence of 2.5% and requires attention during the procedure.
